# Force-Induced Unfolding of Fibronectin in the Extracellular Matrix of Living Cells

**DOI:** 10.1371/journal.pbio.0050268

**Published:** 2007-10-02

**Authors:** Michael L Smith, Delphine Gourdon, William C Little, Kristopher E Kubow, R. Andresen Eguiluz, Sheila Luna-Morris, Viola Vogel

**Affiliations:** Laboratory for Biologically Oriented Materials, Department of Materials, Swiss Federal Institute of Technology Zurich, Zurich, Switzerland; Adolf-Butenandt-Institut, Germany

## Abstract

Whether mechanically unfolded fibronectin (Fn) is present within native extracellular matrix fibrils is controversial. Fn extensibility under the influence of cell traction forces has been proposed to originate either from the force-induced lengthening of an initially compact, folded quaternary structure as is found in solution (quaternary structure model, where the dimeric arms of Fn cross each other), or from the force-induced unfolding of type III modules (unfolding model). Clarification of this issue is central to our understanding of the structural arrangement of Fn within fibrils, the mechanism of fibrillogenesis, and whether cryptic sites, which are exposed by partial protein unfolding, can be exposed by cell-derived force. In order to differentiate between these two models, two fluorescence resonance energy transfer schemes to label plasma Fn were applied, with sensitivity to either compact-to-extended conformation (arm separation) without loss of secondary structure or compact-to-unfolded conformation. Fluorescence resonance energy transfer studies revealed that a significant fraction of fibrillar Fn within a three-dimensional human fibroblast matrix is partially unfolded. Complete relaxation of Fn fibrils led to a refolding of Fn. The compactly folded quaternary structure with crossed Fn arms, however, was never detected within extracellular matrix fibrils. We conclude that the resting state of Fn fibrils does not contain Fn molecules with crossed-over arms, and that the several-fold extensibility of Fn fibrils involves the unfolding of type III modules. This could imply that Fn might play a significant role in mechanotransduction processes.

## Author Summary

Cells are embedded within an extracellular matrix that regulates many cellular processes, including stem cell differentiation and cancer progression. Yet the underlying molecular mechanisms that mediate these processes remain unknown. Within the extracellular matrix of cells, super-molecular assemblies of fibronectin are dynamically stretched many times beyond their resting length by cell traction forces. Whether mechanical forces generated by cells can mechanically unfold fibronectin has been controversial. Clarification of this issue is important since fibronectin displays a large number of molecular recognition sites whose display might be altered by protein unfolding. Here, we used spectroscopic approaches to visualize whether this extracellular matrix protein is unfolded in cell culture. We show that indeed cell traction forces straighten fibronectin and unfold its modules. Fluorescence resonance energy transfer reveals the extent to which the extracellular matrix unfolds and thus potentially regulates cell signaling processes.

## Abstract 3

The extracellular protein fibronectin stretches because of cell traction forces, resulting in both straightening of an initially modular structure and unfolding of fibronectin type III modules. Thus, cell signaling might be regulated by the extent to which the extracellular matrix unfolds.

## Introduction

Fibronectin (Fn), a major component of the extracellular matrix (ECM) of developing tissues and healing wounds, is a large, dimeric protein consisting of more than 50 repeating subunits (for review see [1–6]). Fn displays a number of surface-exposed molecular recognition sites for cells, including integrin binding sites such as the RGD loop, PHSRN synergy site, and LDV sequence, and binding sites for other ECM components, including collagen, heparin, and fibrin. Together these binding sites provide Fn with a diverse array of scaffolding and cell recognition functions. In addition, a number of cryptic binding sites, sequences normally buried in the equilibrium fold of the protein, and surface-exposed binding sites have been proposed to be exposed or deactivated, respectively, as a result of force-dependent conformational change (as reviewed in [2]). It has therefore been hypothesized that in addition to other physical properties of the ECM such as substrate rigidity and matrix composition, matrix unfolding may alter outside-in cell signaling.

**Figure 1:**
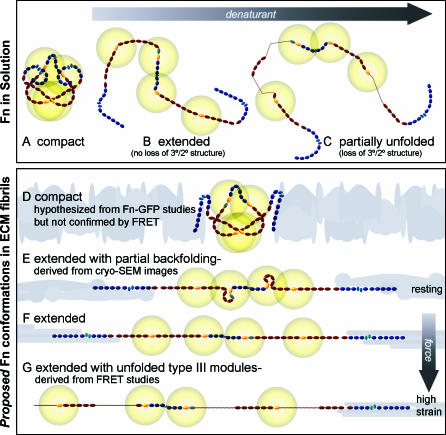
Schematic Sketch of Putative Fn Conformations in Solution and within ECM Fibrils Fn consists of tandem repeats of type I (dark blue ovals), II (narrow, light blue ellipses), and III modules (dark red ovals). Average end-to-end lengths of each module type are drawn to scale using lengths of 2.5 nm for Fn type I [51], 0.7 nm for Fn type II [52], and 3.2 nm for FnIII modules [53]. Two free cysteines are present on each monomer within FnIII_7_ and III_15_ (yellow). Energy transfer between donors and acceptors bound to free cysteines are limited to within approximately double the Förster radius (∼12 nm), denoted by gold circles around III_7_ and III_15_. Fn in solution assumes a compact conformation stabilized through ionic interactions between dimer arms (A) [7]. Denaturant destabilizes these ionic interactions, leading to an extended conformation (B), and higher denaturant concentrations lead to loss of tertiary/secondary structure of FnIII modules (C). The quaternary model for fibril elongation [11,12] predicts a compact conformation with crossover of opposing arms in the absence of tension (D). In contrast, high-resolution cryo-scanning electron microscopic images of Fn fibrils [39,40], taken together with our FRET studies, suggest that fully relaxed fibers do not contain the compact quaternary structure, but are composed of Fn in an extended conformation with partial backfolding of its arms upon themselves (nodules; E). Cell-generated tensile forces first extend Fn fibrils (F) and finally unfold FnIII modules (G).

Soluble Fn in physiological buffer has a compact, folded quaternary structure (Figure 1A) stabilized through intermonomer ionic interactions between III_2–3_ of one arm and III_12–14_ of the opposing arm [7]. Low concentrations of chemical denaturants first destabilize these ionic interactions, leading to separation of the crossed-over arms (extended structure; Figure 1B), and increasing denaturant concentrations finally unfold Fn (Figure 1C). Erickson originally proposed that module unfolding constituted the mechanism for fibril extensibility by estimating the free energy of denaturation and extension of Fn type III (FnIII) modules in comparison to the force generated by single myosin or kinesin motor proteins [8]. Yet the conformation of Fn within fibrillar ECM is still debated [8–12]. Two structural models to explain the several-fold, force-induced extension of Fn within fibrils have been proposed:

The quaternary structure model proposes that Fn within fully relaxed fibrils assumes a compact structure with crossed-over Fn arms similar to that found in solution (Figure 1D; [12]). Under the influence of cell traction, tensile force would first separate the Fn arms, and finally align them along the force vector (Figure 1E) with still intact tertiary/secondary structures of individual modules (Figure 1F; [12]). This model postulates that the fibrils break before Fn starts unfolding.

The unfolding model for Fn elongation proposes that fully relaxed fibrils are composed of Fn in an already extended conformation where the dimeric arms are already separated (Figure 1F) and that fibril extension originates from the unfolding of FnIII modules (Figure 1G). Type I and II modules are stabilized by intramodular disulfide bonds, and therefore only FnIII modules can be completely unfolded by force. FnIII modules have been shown to unfold by passing through several intermediate states [13–18] .

While previous fluorescence resonance energy transfer (FRET) studies indicated that cell contractility is sufficient to unfold fibrillar Fn [9,10,19], the alternative quaternary structural model was proposed based on the following two observations. First, a single-molecule Fn–green fluorescent protein (GFP) study [12] showed that the mechanical stabilities of GFP and the FnIII module are similar over the range of pulling velocities tested (50 to 1,745 nm/s). Second, if Fn-GFP is assembled by cells into micro-sized ECM fibers, stretched Fn-GFP fibrils are reported to maintain a constant integrated level of fluorescence even after contraction to 1/3 or 1/4 their starting length [11,20]. Combining this information derived from single-molecule mechanics with spectroscopic data obtained from a densely packed Fn-GFP fiber, it was concluded that FnIII modules do not unfold in Fn fibrils under the influence of cell traction forces since GFP fluorescence was independent of the extent to which the Fn fibril was strained [11,20]. However, it is unclear whether Fn-GFP embedded in a densely packed fiber may have an altered mechanical stability compared to single-molecule Fn-GFP.

Determining whether Fn is indeed unfolded in ECM fibrils in vivo by cell contractile forces is essential to understand the molecular mechanism of Fn fibrillogenesis and whether exposure of the numerous molecular recognition and cryptic sites alters Fn function in a strain-dependent manner [1,2]. Since no experimental techniques were available to directly probe the loss of tertiary/secondary structure of Fn in cell culture, intramolecular FRET was used to gain conformational information [9,10,21–23]. Here, two Fn labeling schemes for FRET were utilized. Amine/cysteine FRET-labeled Fn (amine/cys Fn-DA) was produced by labeling plasma Fn on all four free cysteines within III_7_ and III_15_ (yellow modules in Figure 1) with Alexa 546 acceptors and on seven random amines with Alexa 488 donors. In the second labeling approach, only the free cysteines per Fn dimer were randomly labeled with two donors and two acceptors (cysteine/cysteine FRET-labeled Fn [cys/cys Fn-DA]). The Förster radius of this fluorophore pair is ∼6 nm (from Invitrogen); therefore, energy transfer is limited to within 12 nm of III_7_ and III_15_ (yellow fading spheres in Figure 1). Amine/cys Fn-DA provides sensitivity to the full conformational range since energy transfer can occur between the crossed arms (intermonomer FRET; Figure 1A and 1D) and along the arms (intramonomer FRET; Figure 1B, 1C, and 1E–1G). The crossover of Fn arms in the fully compact conformation brings the fluorophores attached to III_7_ of opposing arms into close proximity (see Figure 1A and 1D). Therefore, cys/cys Fn-DA is sensitive to intermonomer energy transfer in the compact conformation. However, energy transfer between opposing arms cannot occur when the arms are separated [24,25]. The spatially resolved ratio of acceptor to donor intensities (*I*_A_/*I*_D_) was quantified using both labeling schemes within the ECM of living fibroblasts cultured on glass.

## Results

### Cys/cys Fn-DA FRET Is Highly Sensitive to Loss of Quaternary Structure

Cys/cys Fn-DA with an average of 1.8 donors and 1.9 acceptors was generated by labeling the free cysteines in modules III_7_ and III_15_ of isolated human plasma Fn (unlabeled FN [Fn-u]) with a 15-fold molar excess each of Alexa 546 acceptors and Alexa 488 donors. Labeled Fn was verified to be both dimeric and not contaminated with Fn fragments (as confirmed by Coomassie blue staining of samples run in SDS-PAGE gels; Figure S1A). Given the large size of Fn, our approach utilizes multiple fluorophores on each Fn dimer such that the cumulative *I*_A_/*I*_D_ is sensitive to large conformational changes. Unlike single donor/acceptor pair–labeled proteins, which can be used as nanoscopic rulers [26,27], donor–acceptor distances can thus not be calculated. Confocal microscopy was used to acquire donor, acceptor, and differential interference contrast (DIC) images, and ratiometric *I*_A_/*I*_D_ images were generated by averaging, background subtraction, thresholding, and color-coding. Intensities were measured simultaneously on two photomultiplier tubes (PMTs) for each pixel in a given field of view, with 12-nm bandwidths over acceptor and donor emission peaks.

Energy transfer was first quantified for known conformations of cys/cys Fn-DA in solution with varying concentrations of guanidine hydrochloride (GdnHCl). FRET was independent of the concentration of Fn labeled with Alexa 488 and Alexa 546 (Fn-DA) in the range from 0.05 to 0.25 mg/ml, indicating that intermolecular FRET did not occur in solution (data not shown), and solution *I*_A_/*I*_D_ measurements were found to be constant over a large range of measured intensities (Figure S1B). Up to 6 M GdnHCl did not impact the FRET efficiency of a similar fluorophore pair (Alexa 488/Alexa 594; [28]), suggesting denaturation measurements are not affected by GdnHCl.

**Figure 2:**
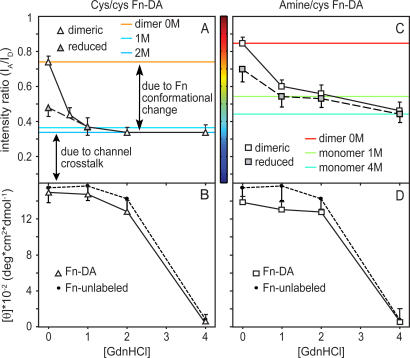
Fn-DA Unfolding in Denaturant Solution Probed by FRET and Circular Dichroism Dimeric Fn-DA (open symbols) or monomeric Fn-DA generated by reduction in 50 mM DTT for 60 min (shaded symbols) with donors and acceptors labeled exclusively on cysteines (cys/cys Fn-DA; A and B) or on amines and cysteines (amine/cys Fn-DA; C and D) was unfolded in 0, 0.6, 1, 2, and 4 M GdnHCl. *I*_A_/*I*_D_ was quantified by calculating the ratio of measured acceptor and donor intensities using 12-nm bandwidths from 566–572 nm and 514–526 nm, respectively (A and C). Mean residue ellipticity was measured for labeled Fn-DA and Fn-u (black circles) in GdnHCl (B and D). Horizontal colored lines for dimeric amine/cys Fn-DA in 0 M GdnHCl, monomeric amine/cys Fn-DA in 1 and 4 M GdnHCl, and dimeric cys/cys Fn-DA in 0, 1, and 2 M GdnHCl are shown based on an artificial color code ranging from 0.05 to 1.0 *I*_A_/*I*_D_ units that is identical to that used in Figures 3–6. Bar width corresponds to the standard deviation of the mean values from three different measurements in each denaturant concentration.

Compactly folded dimeric cys/cys Fn-DA in phosphate-buffered saline (PBS) showed energy transfer (*I*_A_/*I*_D_ = 0.74) that was significantly attenuated in the extended conformation in 1 M GdnHCl (*I*_A_/*I*_D_ = 0.37) and reached a baseline of *I*_A_/*I*_D_ = 0.34 in both 2 M and 4 M GdnHCl (Figure 2A). Monomeric Fn-DA (Figure S1A) reduced in 50 mM DL-dithiothreitol (DTT) [29] was used to eliminate intermonomer energy transfer. Reduced Fn-DA showed intermediate energy transfer in PBS (*I*_A_/*I*_D_ = 0.48); however, *I*_A_/*I*_D_ values for monomeric and dimeric cys/cys Fn-DA in 1, 2, and 4 M GdnHCl were identical. *I*_A_/*I*_D_ does not fall to zero in 4 M GdnHCl although Fn-DA is almost completely unfolded (Figure 2B). This baseline ratio does not reflect residual conformational sensitivity, but instead reflects spectral crosstalk from direct excitation of the acceptor with 488-nm light and bleed emission from the donor into the acceptor detection window.

**Figure 3:**
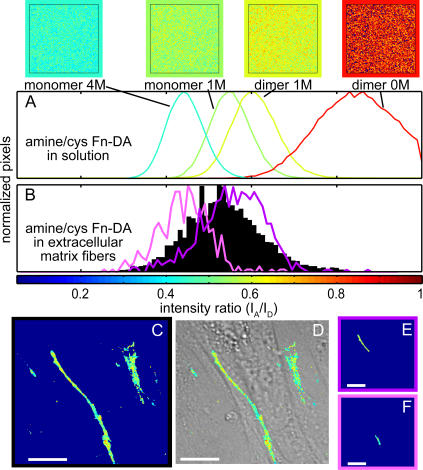
Fn Conformation in Matrix Fibrils Spatial ratiometric images and histograms of all pixels within each field of view are shown for dimeric amine/cys Fn-DA in 0 and 1 M GdnHCl and monomeric amine/cys Fn-DA in 1 and 4 M GdnHCl (A). Amine/cys Fn-DA was added to the culture medium of fibroblasts for 24 h, and excess Fn-u was added to suppress intermolecular energy transfer. Confocal microscopic images of acceptor and donor peak intensities taken 1 μm above the glass–cell interface were background subtracted, averaged, and thresholded, and the *I*_A_/*I*_D_ ratiometric image of acceptor to donor was color-coded within the range of 0.05 to 1.0. A histogram (B) for all pixels of amine/cys Fn-DA–containing ECM (C) and an overlay of *I*_A_/*I*_D_ on the DIC image (D) are shown in a region in which the matrix showed a transition from low to intermediate *I*_A_/*I*_D_ within a single Fn fiber. Histograms are overlaid in (B) for regions of extended (E; purple) and unfolded Fn (F; pink). Histograms were generated with 0.01-ratio-unit bin widths. Scale bars = 25 μm.

To test whether the above baseline *I*_A_/*I*_D_ for monomeric cys/cys Fn-DA in PBS results from the reported artificial dimerization of Fn monomers stabilized through intermonomer ionic interactions [7,30], monomeric labeled Fn-DA was incubated with monomeric Fn-u, since artificial dimerization of one monomer of Fn-u with another monomer of Fn-DA should result in a loss of *I*_A_/*I*_D_ relative to a solution of pure Fn-DA in PBS. Incubation of monomeric Fn-DA with increasing concentrations of Fn-u in PBS, but not in 1 M GdnHCl, where dimerization is inhibited by denaturant, resulted in a gradual decrease in the solution *I*_A_/*I*_D_ (data not shown), suggesting dimerization of reduced monomers affected measurements in PBS. Thus, energy transfer with cys/cys Fn-DA only occurs between dimer arms, and cys/cys Fn-DA is well suited to probe for the overlap of Fn arms or the compact, folded quaternary structure of Fn in ECM fibrils.

Circular dichroism (CD) spectra of cys/cys Fn-DA at 228 nm—an indication of β-sheet content—was measured for Fn-DA to determine whether labeling of Fn with Alexa dyes prevented refolding of Fn after labeling (Figure 2B). The mean residue ellipticity of cyc/cys Fn-DA indicated that Fn-DA refolding was not impacted by fluorophore labeling within experimental error (Figure 2B): measurements in 0 M GdnHCl were not statistically different from measurements on Fn-u and were similar to previously published data [24,31,32].

### Amine/cys Fn-DA FRET Is Sensitive to Loss of Both Quaternary and Tertiary Structure

Amine/cys Fn-DA was produced through site-specific labeling of Alexa 546 on III_7_ and III_15_ and random labeling of Alexa 488 on amines. The number of donors per Fn dimer must be large enough so that a randomly attached donor is present within ∼12 nm of III_7_ or III_15_—a requirement for intramonomer energy transfer. However, excess donors increase background fluorescence from donors that do not form FRET pairs and may cause donor fluorescence self-quenching [33]. Control experiments using donor-labeled Fn showed that only donor-labeled Fn with more than ∼10 donors per Fn dimer had an increased fluorescent yield in denaturant relative to PBS, which indicates fluorescence self-quenching (data not shown). For these reasons, we used amine/cys Fn-DA with 3.9 acceptors and 7.0 donors generated with 30- and 65-fold molar excesses of acceptors and donors, respectively.

The compact quaternary conformation of amine/cys Fn-DA in PBS revealed strong energy transfer (*I*_A_/*I*_D_ = 0.85; Figure 2C). The extended conformation in 1 M GdnHCl had reduced energy transfer (*I*_A_/*I*_D_ = 0.61), while partial unfolding of the extended structure in 2 M GdnHCl reduced *I*_A_/*I*_D_ further to 0.56. Significant unfolding of amine/cys Fn-DA in 4 M GdnHCl led to a further reduction to 0.47, indicating amine/cys Fn-DA retains intramonomer energy transfer in 1 and 2 M GdnHCl and may be used to differentiate extended Fn (Figure 1F) from unfolded Fn (Figure 1G). Monomeric amine/cys Fn-DA showed reduced energy transfer in PBS (*I*_A_/*I*_D_ = 0.65) and 1 (*I*_A_/*I*_D_ = 0.55), 2 (*I*_A_/*I*_D_ = 0.53), and 4 M GdnHCl (*I*_A_/*I*_D_ = 0.44) relative to dimeric Fn-DA. Finally, CD spectra of amine/cys Fn-DA at 228 nm indicated that amine/cys Fn-DA refolds after labeling (Figure 2D), and Fn-DA in 4 M GdnHCl was unfolded (Figure 2D). Thus, amine/cys Fn-DA can be used to differentiate between extended and unfolded conformations of Fn.

In order to estimate the *I*_A_/*I*_D_ value below which unfolding occurs, FRET from Fn-DA denatured in solution was compared to the corresponding CD spectra (Figure 2). While the first loss of secondary structure is seen in CD above 1 M GdnHCl and the protein is almost completely unfolded in 4 M GdnHCl, FRET differences remain between the monomeric and dimeric amine/cys Fn-DA solutions even up to 4 M GdnHCl. This is most likely due to occasional crossover of dimer arms during free diffusion in solution, an effect that is far less pronounced in cys/cys Fn-DA solutions because of the reduced number of fluorophores distributed along the Fn arms (the similar values for monomeric and dimeric cys/cys Fn-DA also indicate that the difference in monomeric and dimeric amine/cys Fn-DA in 1 M GdnHCl was not due to DTT but reflected fluorophore separation). Therefore, *I*_A_/*I*_D_ for monomeric amine/cys Fn-DA in 1 M GdnHCl (*I*_A_/*I*_D_ = 0.55) was used here to indicate the onset of module unfolding. The conclusions drawn in this study, however, are not altered in a major way if slight differences exist between the *I*_A_/*I*_D_ values at which secondary structure is lost by chemical denaturation or mechanical unfolding. The data indicate that the unfolding point within fibrils might occur between the *I*_A_/*I*_D_ value taken at 1 or 2 M GdnHCl since only a slight drop in *I*_A_/*I*_D_ was noted between monomeric amine/cys Fn-DA in 1 (*I*_A_/*I*_D_ = 0.55) and 2 M GdnHCl (*I*_A_/*I*_D_ = 0.53).

### Fn Assumes a Broad Range of Conformations in Fibroblast ECM

To determine whether the compact quaternary and unfolded conformations of Fn are present in ECM, cys/cys or amine/cys Fn-DA was next added to a culture medium of human fibroblasts that readily incorporate exogenously added soluble Fn into their matrix fibrils [9,10,34]. At a seeding density of 20 × 10^3^ cells/cm^2^, fibroblast ECM was three-dimensional after 24 h and ranged from ∼4 to 10 μm in thickness. Cells were either fixed in 3% formaldehyde and imaged under PBS [10] or imaged live under PBS. Data were similar between fixed and live samples (data not shown). Photobleaching of fluorophores was routinely tested by acquiring consecutive images of a random field of view and plotting histograms of the differences in acceptor (Figure S1C and S1D) and donor intensities (Figure S1E and S1F) on a pixel by pixel basis between consecutive images. Finally, the ratio of amine/cys or cys/cys Fn-DA to Fn-u was titrated over a wide range to determine the percentage of Fn-DA above which intermolecular FRET occurred. This analysis demonstrated that 10% amine/cys Fn-DA is not contaminated by intermolecular FRET (Figure S1G). However, greater than 10% amine/cys Fn-DA led to progressively increased mean *I*_A_/*I*_D_ values in fibrils. In contrast, mean *I*_A_/*I*_D_ measurements in ECM fibers using cys/cys Fn-DA were not dependent upon the ratio of cys/cys Fn-DA to Fn-u at ratios below 50% Fn-DA. This is consistent with the reduced number of fluorophores with this approach (∼4) relative to amine/cys Fn-DA (∼11).

To assess the extent to which *I*_A_/*I*_D_ measurements represent conformational variability versus instrument noise, FRET histograms of ECM were compared to those taken from Fn-DA in solution (Figure 3A). The width of histograms derived from solution measurements could be reduced without affecting population means by using higher concentrations of Fn-DA, higher excitation power, or multiple acquisitions and subsequent averaging of each pixel, indicating that noise is the predominant contributor to the width of histograms from solution measurements. Noise contributes significantly to the variability of individual ratiometric measurements as signals in the numerator (*I*_A_) and denominator (*I*_D_) are acquired by separate PMTs (see Text S1 and Figure S2). Figure 3 and Video S1 show a histogram (Figure 3B) for all pixels of amine/cys Fn-DA–containing ECM in a fibroblast culture acquired 1 μm above the cell–glass interface (Figure 3C), with an overlay of *I*_A_/*I*_D_ on the DIC image (Figure 3D). The 80-μm Fn fiber at the interface between two cells in the confluent culture transitions from an extended conformation at the upper left edge (Figure 3D; purple outline) through a region with unfolded Fn (Figure 3E; pink outline). While noise contributes significantly to the broadening of FRET data, the different colors seen in the ECM images in every field of view are not just due to instrument noise. Individual fibers with higher or lower mean *I*_A_/*I*_D_ values can be distinguished, and their histograms are narrower than the histogram derived from the entire field of view (Figure 3D and 3E; each region >100 pixels), thereby demonstrating that different Fn conformations do coexist.

To test for the presence of the fully compact solution structure of Fn, cys/cys Fn-DA was incorporated into a 24-h fibroblast ECM. Figure S3A shows a ratiometric confocal slice 4 μm above the glass–ECM interface. Cell extraction with Triton X-100 (Figure S3B) or alternatively Rho kinase (ROCK) inhibition with Y-27632 (Figure S3C) were used to inhibit cellular contractile forces, both causing matrix relaxation [10,20]. Histograms for all pixels in three random fields of view each from three separate experiments are plotted in Figure S3D. Some regions of cyc/cys Fn-DA matrix were composed of Fn with some partial backfolding of the monomeric arms upon themselves since *I*_A_/*I*_D_ values were higher than that measured for the extended structure but lower than that measured for the fully compact solution structure (see Discussion). These data confirm that the compact solution structure of Fn was never achieved in fibrillar Fn even after release of cell traction forces.

**Figure 4:**
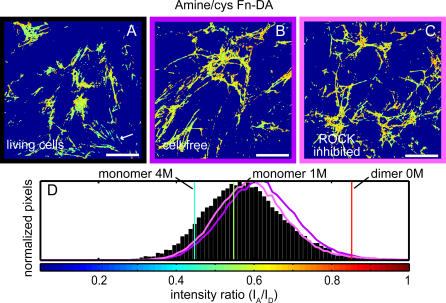
Matrix Refolding after Cell Extraction or ROCK Inhibition Amine/cys Fn-DA and excess Fn-u were added to the culture medium of fibroblasts for 24 h. Color-coded *I*_A_/*I*_D_ ratiometric images are shown for control cells (A), extracted cell-free matrix (B), and fibroblast cells after 60 min exposure to the ROCK inhibitor Y-27632 (C). Histograms with 0.01-ratio-unit bin widths for all pixels of control (black), cell-free (purple), and ROCK-inhibited matrix (pink) were derived from three random fields of view each from three separate experiments in each group (D). Solution denaturation values for dimeric Fn-DA in 0 M GdnHCl and monomeric Fn-DA in 1 and 4 M GdnHCl are shown as red, green, and blue lines, respectively. Scale bars = 50 μm.

To test for the presence of unfolded Fn, amine/cys Fn-DA was next incorporated into a 24-h fibroblast matrix. Figure 4A shows a representative ratiometric image acquired 4 μm above the glass–ECM interface. On average, Fn was in the extended conformation in fibroblast culture (*I*_A_/*I*_D_ = 0.59 ± 0.14); however, unfolded Fn fibers with mean *I*_A_/*I*_D_ < 0.55 were present within all imaged confocal slices. These regions were not randomly dispersed, but instead whole fibrils encompassing at least 50 pixels were present (Figure 4A, white arrow). Cell extraction (Figure 4B) or inhibition of ROCK (Figure 4C) resulted in an increase in *I*_A_/*I*_D_ and a decrease in the presence of unfolded pixels (*I*_A_/*I*_D_ < 0.55). Histograms generated from three random fields of view, each from three separate experiments (Figure 4D), showed that loss of cell contractile force leads to refolding of Fn within matrix fibrils. Finally, only fewer than 1% of pixels had *I*_A_/*I*_D_ values representative of the fully compact solution conformation of Fn. In addition, whole fibrils consisting of at least 50 pixels were never measured with mean *I*_A_/*I*_D_ > 0.75 (data not shown). Since each pixel measurement represents an average over a population of molecules, multiple and distinct conformations may coexist within a single pixel. If a fraction of Fn-DA molecules within each pixel is in the fully compact conformation, they would have to be offset by another fraction of molecules with *I*_A_/*I*_D_ significantly lower than the overall mean value. However, some histograms for all pixels within regions of interest containing individual ECM fibers had both width and median values similar to those measured for Fn-DA in 4 M GdnHCl (Figure 3B and 3F). If these fibers were in fact heterogeneous populations, a small portion of Fn-DA that was not unfolded would have to be offset by Fn-DA that had lower *I*_A_/*I*_D_ than that measured in 4 M GdnHCl. This is unlikely since Fn-DA is almost completely unfolded in 4 M GdnHCl, and these distributions are indicative of a population of Fn molecules with conformational homogeneity. Finally, accumulated FRET between several donors and acceptors cannot differentiate between partial unfolding into structural intermediates and complete unfolding of FnIII modules. However, more than twice as many partially unfolded FnIII modules as completely unfolded modules would be needed to account for a given increase in end-to-end length. Partial unfolding into an intermediate conformation where two β-strands are separated would only increase the module end-to-end length by ∼1/3 of the increase due to complete unfolding [13,14,16,18].

### Exogenous Strain Application to Cell-Free Fn Matrix Can Tune Fn Conformation

**Figure 5:**
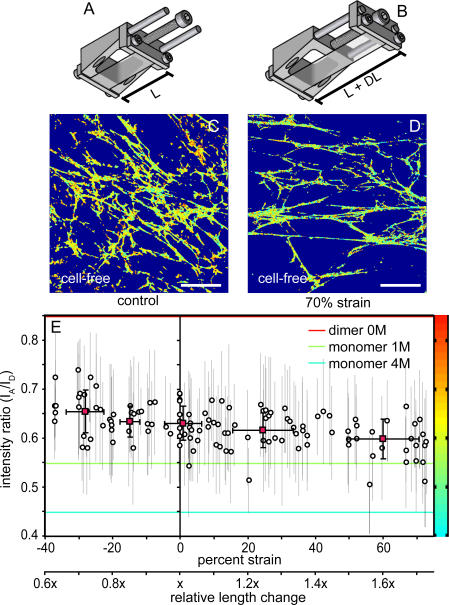
Fn Conformation after Application of Exogenous Strain A schematic of the strain device is shown in the relaxed configuration with length L before (A) and length L + DL after (B) application of strain. PDMS sheets were covalently modified with Fn-u as described in Materials and Methods, and fibroblast cells were cultured for 24 h in the presence of amine/cys Fn-DA and excess Fn-u. Cells were extracted in mild detergent. Color-coded *I*_A_/*I*_D_ ratiometric images are shown for a field of view without application of stretch (C) and after application of 70% elongation strain with 28% transverse compression (D). Region of interest analysis on individual fibrils was used to determine the impact of elongation on *I*_A_/*I*_D_ on a per fibril basis (circles, mean ± standard deviation), and binned averages were calculated for fibrils between −37% and −20%, −20% and −10%, −10% and 10%, 10% and 40%, and 40% and 73% strain (red squares, mean ± standard deviations) (E). Abscissa is also plotted as relative length change. Solution values for dimeric Fn-DA in 0 M GdnHCl and monomeric Fn-DA in 1 and 4 M GdnHCl are shown as horizontal red, green, and blue lines, respectively. Scale bars = 50 μm.

Contractility inhibition studies showed that matrix refolding occurs after loss of cell generated forces (Figure 4); however, the relationship between FRET changes and changes in fibril length were unknown. We therefore designed a strain device (see also Text S1 and Figure S4) to stretch denuded Fn matrix fibrils attached to a stretchable substrate, which allowed us to calibrate the relationship between mechanical strain and FRET. Schematic diagrams of the strain device before and after strain application are shown in Figure 5A and 5B. Figure 5C and 5D show representative ratiometric images of denuded amine/cys Fn-DA matrix on unstretched poly(dimethylsiloxane) (PDMS) and on PDMS after a 1.7-fold increase in length (70% elongation, 28% transverse shortening), respectively. *I*_A_/*I*_D_ values for individual amine/cys Fn-DA–containing fibrils were measured for up to six fibrils per field of view from 26 fields of view in six experiments, and the strain was calculated based on their angular orientation with respect to the strain axis. The individual fibril mean *I*_A_/*I*_D_ values are plotted versus the calculated strain in Figure 5E. Relative strain of individual fibrils ranged from relaxation to 3/5 the starting length (−40% strain) to a 1.7-fold stretch (73% strain). Cell-free control fibrils measured prior to stretch or relaxation (*I*_A_/*I*_D_ = 0.62 ± 0.03) were composed mainly of Fn in the extended conformation, but ranged from unfolded (*I*_A_/*I*_D_ lower limit = 0.54) to extended with some backfolding of the monomeric arms (*I*_A_/*I*_D_ upper limit = 0.69). Therefore, denuded Fn matrix assembled on PDMS was similar in conformation to denuded Fn matrix on Fn-coated glass. Mean *I*_A_/*I*_D_ dropped to 0.59 ± 0.04 for fibrils stretched by more than 1.4-fold (>41% strain), while relaxation to less than 4/5 the starting length (−21% strain) resulted in a statistically significant increase in *I*_A_/*I*_D_ to 0.65 ± 0.04. Thus, the greater than 2-fold length change required to traverse from −21% to 60% strain resulted in a shift in mean fibril *I*_A_/*I*_D_ of only ∼0.07, a difference that is less than the total range of *I*_A_/*I*_D_ (0.54 to 0.69) measured for control cell-free fibrils prior to stretch or relaxation. From these data we conclude that matrix fibrils in a typical field of view containing ∼15–20 confluent cells are heterogeneously stretched by a more than 2-fold difference in length and that ECM strain covers a wider range than was previously appreciated. Control experiments using cys/cys Fn-DA did not show a strain response, even if stretched by up to 75% (Figure S5), indicating that cys/cys Fn-DA is insensitive to differentiating between extended and unfolded Fn.

**Figure 6:**
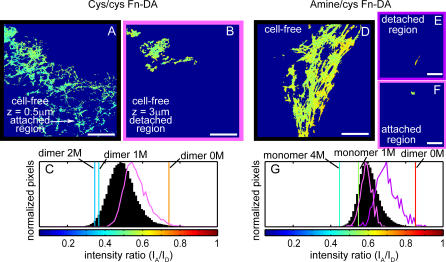
Fully Relaxed Fn-DA Does Not Assume the Fully Compact Conformation Cys/cys Fn-DA (A–C) or amine/cys Fn-DA (D–G) was incorporated into fibroblast matrix on Fn-u that was adsorbed to plasma cleaned PDMS, and after cell extraction the substrate was relaxed to 4/5 (A and B; 3.7% transverse stretch) or 3/5 the starting length (D–F; 10% transverse stretch). *I*_A_/*I*_D_ ratiometric images of cys/cys Fn-DA–containing matrix are shown at the PDMS–ECM interface (A), where a portion of the cell-free fibers are still attached to the substrate, and from the same field of view but acquired 3 μm above the PDMS surface (B), where the strain-free Fn mat randomly diffused around its points of attachment to the underlying ECM. Histograms are shown for all pixels within the field of view at the substrate (C; black) and from the upper, strain-free confocal slice (C; pink). An *I*_A_/*I*_D_ ratiometric image of amine/cys Fn-DA is shown with both detached (E) and still-attached (F) regions of matrix within the same confocal slice. Region of interest analysis was used to generate histograms (G0 for all pixels within the detached (E and G; purple) and attached (F and G; pink) regions of matrix, which were overlaid on a histogram of all pixels in the field of view (black). Scale bars = 50 μm.

### Even in Fully Relaxed Matrix Fibrils, Fn Does Not Assume the Compact Solution Conformation

To test the primary assumption of the quaternary structure model (Figure 1D), which proposes that Fn assumes a compact (solution) conformation in the resting state of fibrils [12], fibroblast matrix composed of cys/cys Fn-DA (Figure 6A–6C) or amine/cys Fn-DA (Figure 6D–6G) was grown on Fn-u adsorbed onto PDMS sheets. Without covalent attachment of Fn-u to PDMS, two methods could be used to partially detach the denuded Fn matrix from the substrate. We found that 10%–20% strain resulted in detachment of large regions of de-cellularized ECM (Figure S6). To circumvent the straining step, fibroblast matrix was also assembled on Fn-u adsorbed to prestretched PDMS and partially detached by relaxation of the denuded matrix to from 4/5 (Figure 6A–6C; 3.7% transverse stretch) to 3/5 (Figure 6D–6G; 10% transverse stretch) the starting length. Figure 6A shows a ratiometric image just above the PDMS substrate, while Figure 6B shows an image from the same field of view but acquired 3 μm above the substrate. This relaxed Fn mat was loosely attached to the matrix shown in Figure 6A, but it randomly diffused in the field of view around its points of attachment. Both detachment schemes resulted in *I*_A_/*I*_D_ means for fully relaxed fibrils containing cys/cys Fn-DA (Figure 6C) or amine/cys Fn-DA (Figure 6G) that varied from 0.48 to 0.58 and 0.68 to 0.75, respectively. The percentage of fully compact pixels never exceeded 2% using cys/cys Fn-DA, while at most 6% of pixels were fully compact using amine/cys Fn-DA. These data show that complete relaxation of cell-free fibers leads to a complete loss of unfolded Fn and a complete absence of fully compact Fn. Therefore, we conclude that extension of Fn fibers by cell traction forces occurs through both straightening of partially backfolded monomer arms (residual quaternary structure) (Figure 1E; see also Discussion) and unfolding of FnIII modules (Figure 1G). Moreover, unfolded conformations, extended molecules with intact tertiary structure, and extended conformations with some residual quaternary structure dynamically coexist within the ECM.

## Discussion

The ECM contains numerous super-molecular fibrillar assemblies of proteins that display a remarkable range of end-to-end extensions under physiologically relevant forces. Indirect evidence from single-molecule atomic force microscopy [17] and cryptic epitope exposure detected by monoclonal antibody binding [35] suggested that Fn fibers in the ECM are unfolded by cell traction forces; however, this possibility was recently debated [8–12,20]. We used two different FRET labeling schemes to test for the presence of the compact solution conformation in fibrils, resulting from dimer arm overlap, and the unfolded conformation of Fn within ECM (Figure 1). Intramolecular energy transfer using cys/cys Fn-DA was shown in solution measurements to be sensitive mainly to dimer arm overlap (Figure 2). In contrast to cys/cys Fn-DA, amine/cys Fn-DA is also sensitive to both the extended and unfolded conformations of Fn (Figure 2), thus confirming earlier findings [9,10,24]. FRET from both labeling schemes was used to demonstrate that the compact solution conformation of Fn is not present within fibroblast matrix in the presence of cell contractile forces, after inhibition of cell contractility (Figures 3, 4, and S3), or even in strain-free fully relaxed Fn fibrils (Figures 6 and S6). Partially unfolded Fn, however, is present in ECM fibers of live fibroblast cultures, and some FnIII modules refold after blocking cell contractility (Figure 4). Unfolded Fn is absent in fully relaxed fibers (Figures 6 and S6).

### How to Reconcile the Controversy Derived from Fn-GFP and FRET Data?

**Figure 7:**
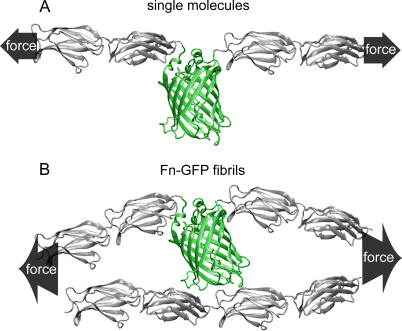
Restricting GFP Movement if Embedded in a Fn Fibris Cartoons of FnIII modules (gray) and GFP (green) were generated to scale using Tachyon renderer and implemented in VMD version 8.5 to illustrate the degree of protrusion from the central axis of an extended Fn-GFP molecule. In single-molecule pulling experiments, GFP has the freedom to align along the direction of force (A). If embedded into a densely packed Fn fiber that is stretched by mechanical force, the rotational motion of GFP is restricted (B), which could lead to unfolding along a different pathway.

Since Fn contains more than 54 domains and is ∼130 nm long in the extended conformation without unfolding of its FnIII modules [36], a 3- to 4-fold change in end-to-end length could theoretically occur through either quaternary structural change (Figure 1D–1F) or secondary/tertiary structural change (Figure 1F and 1G). Erickson and collaborators showed that Fn-GFP chimeric proteins embedded within ECM fibrils retained constant levels of fluorescence after fibrils were cut [20], while single-molecule force measurements revealed that individual GFP and FnIII modules (Figure 7A) were similarly resistant to mechanical unfolding [12]. In consideration of this experimental evidence together with the FRET data presented here that FnIII modules are indeed unfolded within fibroblast ECM, we conclude that Fn-GFP embedded within tightly packed Fn fibrils has an increased mechanical stability compared to single-molecule Fn-GFP as used for the atomic force microscope studies for the following reasons. While FnIII modules are ellipsoidal in shape with a 2- to 2.5-nm cross-sectional diameter, GFP is a significantly bigger molecule. It forms a cylindrical 4-nm-long β-barrel that protrudes into the space surrounding the string of FnIII modules and might distort the otherwise parallel alignment of Fn molecules within fibrils. The lateral pressure imposed by the adjacent Fn molecules on the GFP barrel if mechanically strained is likely to reduce its freedom of motion, and by minimizing local distortions, its tilt angle with respect to the strain axis might be changed as well (Figure 7B). These and other factors might contribute to the possibility that GFP embedded in densely packed fibers submitted to tensile force unfolds along a different unfolding pathway. Although Fn-GFP fibers tested by Erickson and colleagues contracted up to 1/4 their starting length once cut [11,20], we have recently determined that manually deposited Fn fibrils can be extended 5- to 6-fold from their resting state before they begin to break (W. C. L., M. L. S., U. Ebneter, V. V., unpublished data). Accordingly, we cannot exclude that fibrillar GFP-Fn might still lose its fluorescence if fibrils are stretched more than 4-fold.

### Structural Model of Fibril Extension by Tensile Force

Although FRET data showed that the compact solution conformation of Fn does not exist in ECM fibers, cys/cys Fn-DA from fully relaxed fibrils indicates some minor quaternary structure that disappears in strained fibrils. Bending in hinge regions, for instance between modules I_1–5_ and III_2_ [37] or III_12–14_ [38], could permit backfolding of a monomer arm upon itself, thus bringing otherwise distant Fn segments into close proximity and increasing energy transfer. Although the FRET approach presented here is not capable of differentiating between these different structures, high-resolution cryo-scanning electron microscopic images of cell-derived Fn nanofibrils as small as 5 nm in diameter were either straight or contained nodules 10–15 nm in diameter [39,40]. Smooth nanofibrils were attached at each end, while fibers decorated with nodules had one free end and thus more likely represented fully relaxed fibrils. In addition, fibrils that were free at one end were as narrow as 5 nm in diameter, while fully relaxed fibers consisting of fully compact Fn should have a diameter of ∼35 nm. From these cryo-scanning electron microscopy data we speculate that a partially backfolded structure, as sketched in Figure 1E with artistic freedom, may represent the fully relaxed conformation of Fn in ECM. However, the structure of Fn in fully relaxed fibers as well as the locations and extent of crosslinking and amount of axial offset between adjacent Fn molecules in fibrils remain to be determined.

Finally, unfolding of FnIII modules occurs through rupture of backbone hydrogen bonds, leading to a peeling away of the β-strands (for review see [2]). Therefore, Fn extension is not a continuous response to tension, but rather an energy-dissipating process with major hysteresis between the unfolding and refolding trajectories [17]. The word “extensibility” was thus used here to describe Fn elongation in place of “elasticity.”

### Physiological Implications of the Existence of Unfolded FnIII Modules

FRET is sensitive to unfolding of modules within less than 12 nm of III_7_ or III_15_. Therefore, partial or full elimination of FRET by tensile force indicates unfolding within modules III_13–14_, III_V_ (present on one monomer of plasma Fn), III_5–6_, or III_8–9_, with possible minor contributions to energy transfer from FnIII modules at the Förster limit (III_4_, III_10_, and III_12_). Based on experiments done with the strain device, the conformational differences seen in a random field of view correspond to a roughly 2-fold difference in Fn end-to-end extension. Each completely unfolded FnIII module would lengthen from ∼3.2 nm to an extended contour length of ∼28.5 nm [17], and an extended Fn molecule estimated to be 130 nm in length [36] could double in length by complete unfolding of only ∼5–6 FnIII modules per Fn molecule.

Not only does Fn end-to-end extensibility contribute to the mechanical resiliency of tissues, but strain could furthermore alter Fn function through unfolding of FnIII modules into partially unfolded intermediate conformations with deactivated or newly exposed molecular recognition sites [1,2]. For instance, steered molecular dynamics simulations predicted that a major intermediate state exists for FnIII_1_ or III_2_ along the force-induced unfolding trajectory [14]. By peeling off two β-strands, force unmasks an analog of anastellin, a proteolytic fragment of module III_1_, that as an isolated peptide inhibits metastasis and angiogenesis but promotes fibrillogenesis [41,42]. Steered molecular dynamics simulations also showed that an increase in the distance between the loop-exposed integrin-binding RGD sequence on module III_10_ and the partner PHSRN synergy site on III_9_, which favors binding of the integrin α_5_β_1_ over α_v_β_3_ [43,44], occurs prior to unfolding of the respective FnIII modules. However, these and other hypotheses that state that the ECM might be involved in mechanotransduction processes are only physiologically relevant if Fn fibrils partially unfold. In addition, molecular recognition sites might also be buried within the nodules and become exposed upon their disappearance. It is thus important to note that the conformational distribution across each field of view within a living cell culture was rather heterogeneous at the length scale of cells and always included a fraction of fibers or fibrillar sections that presented *I*_A_/*I*_D_ values indicative of partially unfolded Fn. Living cells are thus surrounded by and potentially respond to a broad range of fibrillar Fn conformations. Future research is now needed to elucidate the hypothesized physiological importance of partially unfolded Fn. It also remains to be clarified whether module unfolding plays a role in the force-induced fibrillogenesis of Fn and thus only occurs heterogeneously within unfolded regions of the ECM [35,41,45–47].

### Conclusion

Force has a pervasive influence on the behavior of tissues, cell aggregates, and individual cells. However, the molecular motifs that convert force into biochemical signals thus permitting force sensation are only now emerging. The altered functions of non-equilibrium structures of proteins resulting from mechanical tension are beginning to be appreciated as essential to cell function inside the cell cytoplasm, as was recently shown for the signaling molecule p130Cas [48], and in the cell membrane, for instance as a regulator of ion channel function [49]. Here, we show that the extracellular protein Fn is stretched by cell traction forces both from straightening of an initially nodular assembly with quaternary structure and unfolding of FnIII modules. Outside-in cell signaling might thus be regulated not only by the composition and rigidity of the matrix, but also by the extent to which it is unfolded. These results contribute to our understanding of Fn's remarkable mechanical properties and serve as an impetus for future elucidation of the biological importance of mechanically regulated cryptic epitope exposure on Fn.

## Materials and Methods

### Fn labeling for FRET.

Fn isolated from human plasma (see Text S1) was doubly labeled with Alexa 488 and Alexa 546 (Molecular Probes, http://probes.invitrogen.com/) as FRET donors and acceptors, respectively, using two different labeling schemes based on established protocols [24,25]. To produce cys/cys Fn-DA, Fn was site-specifically labeled exclusively on buried cysteines within modules III_7_ and III_15_ of each dimer arm. Isolated plasma Fn at ∼1 g/l in PBS was denatured in an equal volume of 8 M GdnHCl and incubated for 2 h with a 15-fold molar excess each of Alexa 546 maleimide and Alexa 488 maleimide. Fn-DA was separated from free dye by size exclusion chromatography (PD-10 Sephadex, Amersham, http://www.amersham.com/). Western blot analysis was used to determine the point at which Fn fragments contaminated the eluent (data not shown). Fn was also labeled to produce amine/cys Fn-DA with donors and acceptors located on random amines and cysteines, respectively. Fn at ∼1 g/l in PBS was denatured in an equal volume of 8 M GdnHCl and incubated for 1 h with a 30-fold excess of Alexa 546 maleimide. Acceptor-labeled Fn was separated from free dye by overnight dialysis (Slide-a-lyzer dialysis cassette, 10,000 MW cutoff; Pierce, http://www.piercenet.com/) with three changes of amine labeling buffer (PBS with 0.1 M NaHCO_3_ [pH 8.5]). Subsequently acceptor-labeled Fn was incubated with a 70-fold excess of Alexa 488 succinimidyl ester for 1 h. Fn-DA was separated from free dye using a PD-10 column equilibrated with PBS. The labeling ratio of donors to acceptors per Fn dimer was determined by measuring the absorbances of Fn-DA at 280, 496, and 556 nm and using published extinction coefficients for dyes and Fn. Fn-DA was stored with 10% glycerol at −20 °C until needed and used within 5 d of thawing and storage at 4 °C.

### Analysis of labeled proteins in solution.

*I*_A_/*I*_D_ was measured for monomeric or dimeric Fn-DA freely diffusing in solution in different concentrations of denaturant using the same microscopic setup. Small chambers were made by cleaning a 0.17-mm-thick coverslip, 1 × 6 cm PDMS spacers cut from 0.25-mm-thick sheets (Specialty Manufacturing, http://www.specmfg.com/), and a microscope slide in 2% PCC-54 (Sigma-Aldrich, http://www.sigmaaldrich.com/), 70% ethanol, and finally deionized water. Parts were dried, and PDMS strips were placed on the microscope slide to make small channels. The coverslip and the exposed glass between the PDMS spacers were coated with 4% BSA (Sigma-Aldrich) for 1 h to prevent Fn-DA binding to chamber surfaces. BSA-coated surfaces were rinsed with water and dried under filtered air. Finally, the coverslip was placed onto the PMDS strips to make parallel channels between the coverslip and microscope slide. Fn-DA at 0.1 to 0.2 g/l with or without 0 to 4 M GdnHCl or 50 mM DTT in PBS (Sigma-Aldrich) was then drawn into the chambers by capillary forces. Fn-DA in solution within the channels was imaged through the coverslip.

### Circular dichroism.

CD spectra were measured on a Jasco (http://www.jascoinc.com/) model J-715 spectropolarimeter with temperature control using Fn-DA at a concentration *c* that varied from 0.1 to 0.2 g/l. Mean residue ellipticity, [θ], presented in units of degrees × square centimeters/decimole, was calculated from the observed ellipticity, θ_obs_, measured in a cell with path length *l* by

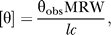
where a value of 108 was used for the mean residue molecular weight, MRW. Measurements of Fn-DA were made in duplicate, while measurements of Fn-u were made in triplicate at 20 °C.

### Cell culture.

Primary human dermal fibroblast cells derived from foreskins (PromoCell, http://www.promocell.com/) were maintained for less than eight passages in Fibroblast Growth Medium plus Supplement (PromoCell). For *I*_A_/*I*_D_ measurements, the growth medium was switched to Dulbecco's modified Eagle's medium (DMEM) plus 10% newborn calf serum (NCS; Invitrogen, http://www.invitrogen.com/), since high serum was found to shorten the culture time necessary for production of a three-dimensional matrix (data not shown). Eight-well Lab-Tek chambers (Nalgene Nunc, http://www.nalgenunc.com/) were coated with 0.03 g/l Fn-u in PBS for 1 h prior to cell seeding at 20 × 10^3^ cells/cm^2^. Cells were allowed to adhere for 30 min, after which the medium and unbound cells were removed and replaced with DMEM plus 10% NCS, 0.005 g/l Fn-DA, and 0.045 g/l Fn-u. Cells were incubated for 24 h before imaging. Samples to be fixed were first washed two times with warm PBS and then treated with 3% formaldehyde for 20 min. Samples were imaged under PBS.

### Strain device.

A custom strain device was manufactured from stainless steel (Figure S3), and cells were grown on modified stretchable PDMS surfaces. PDMS sheets (0.25 mm thick), cut into 5 by 1.7 cm rectangles, and PDMS rings, 2 mm thick with 1.0-cm inner and 1.6-cm outer diameters, were cleaned in PCC-54, ethanol, and finally water. After drying, PDMS rings were placed in the middle of the PDMS sheets, and the PDMS was treated with air plasma (PDC-32G, Harrick Scientific, http://www.harricksci.com/) at 250 mbar for 30 s to render the surface hydrophilic. For noncovalent attachment of Fn-u to the surface, plasma-treated surfaces were used. For covalent attachment of Fn-u, amino groups were attached to the plasma-treated surface with 3-aminopropyltriethoxysilane vapor in an evacuated chamber for 1 h. Vapor-based silanization resulted in significantly lower surface fluorescence than solution-based silanization (data not shown), as has been previously reported [50]. After silanization, 0.125% glutaraldehyde (Sigma-Aldrich) was added and the sample incubated for 20 min. Finally, the surface was rinsed with water and dried prior to assembly of the strain device.

All metal strain device parts were autoclaved prior to assembly within a tissue culture hood. PDMS sheets were clamped into the strain device, and the culture surface within the PDMS ring was incubated with 0.03 g/l Fn-u for 1 h. Unreacted glutaraldehyde was quenched by incubation with DMEM plus 10% NCS for 20 min at 37 °C. Then 20 × 10^3^ fibroblast cells/cm^2^ were seeded onto the PDMS surface and allowed to adhere for 30 min prior to removal of unbound cells and replacement with DMEM plus 10% NCS, 0.005 g/l Fn-DA, and 0.045 g/l Fn-u. After 24 h, cells were washed two times in 37 °C PBS and extracted, leaving behind the detergent-insoluble Fn matrix, with a 5-min incubation in 0.5% Triton X-100 plus 20 mM NH_4_OH in PBS (pH ∼9.7). The strain device with attached cell-free matrix was then imaged. Three separate fibril detachment experiments were performed with both Fn labeling approaches, while four and six separate stretching experiments were performed with amine/cys Fn-DA or cys/cys Fn-DA, respectively, on Fn-u that was covalently attached to the membrane. During strain application, it was found that mounting the PDMS ring on the PDMS membrane prior to treatment with air plasma most often resulted in the PDMS ring sliding on the membrane surface during stretching. Strain samples were discarded if the ring stuck to the membrane, thus leading to inhomogeneous strain application, or leaked culture medium.

### Confocal microscopy.

All FRET images were acquired from living, fixed, or denuded acellular samples in LabTek chambers or in the strain device. All images were acquired with an Olympus (http://www.olympus-global.com/) FV1000 confocal microscope with an oil immersion 1.35NA 60× objective. Emitted light from the sample was split with a 50/50 beam splitter, and it was detected with two PMTs. Spectral information was determined using a diffraction grating and slit. Acceptor and donor intensities were detected using 12-nm bandwidths across the donor (514–526 nm) and acceptor (566–578 nm) emission peaks. Images were generally acquired at 512 × 512 pixel resolution for a 212 × 212 μm field of view with a 200-μm pinhole diameter. Acquisition parameters including laser transmissivity and pixel dwell time were adjusted to prevent photobleaching while maximizing detection sensitivity, and PMT voltages were constant at 600 V. Donor, acceptor, and DIC transmission images were taken from multiple regions of each sample. PMT dark current background values were acquired every 30 min.

### Image processing.

All 16-bit images (4,096 relative intensity units) were processed with Matlab (The MathWorks, http://www.mathworks.com/). Images were averaged with 2 × 2 pixel sliding blocks, mean PMT dark current background values were subtracted from donor and acceptor images, and donor images were corrected for light attenuation from the 50/50 beam splitter with a multiplication factor of 1.09. A threshold mask of 100 relative intensity units was applied to both images, and the acceptor image was divided pixel by pixel by the donor for all pixels above threshold intensity values in both channels. Histograms were computed from all data pixels within each field of view using bin widths of 0.01 intensity ratio units, and *I*_A_/*I*_D_ was color-coded within the range of 0.05 to 1.0. Photobleaching of samples was routinely tested by acquiring two or more consecutive images in both the donor and acceptor channels. Consecutive images were averaged and background subtracted. Next, intensity values from the second image acquisition were subtracted from intensity values from the first acquisition on a pixel by pixel basis, and a histogram was generated for the resultant differential intensity image. Data were not included if histograms showed a greater than 5% mean drop in either donor or acceptor differential intensity histograms (as in Figure S1C and S1D).

In some cases, individual fibril mean *I*_A_/*I*_D_ values were calculated for those fibrils that met specific criteria using region of interest analysis. All regions of interest were chosen from donor images where *I*_A_/*I*_D_ was not shown to limit experimenter bias. Fibrils had to be straight without intermediate branch points and have a length to width ratio of at least five, to increase the probability that the central axis of the fibril was a correct indication of the direction of strain. Fibrils had to be at least 15 μm in length and 50 pixels in size (∼8 μm^2^ surface area within the confocal slice) to increase accuracy of the mean intensity ratio estimate. Straight fibrils were considered only if they were connected on each end by larger Fn structures such as branch points or sheet-like aggregates to increase the probability that each end of the fibril was connected to the PDMS substrate. If more than six fibrils in a field of view met these criteria, the six largest fibrils were chosen. The region of interest was drawn around the straight section of fibrils, and the mean plus standard deviation of the intensity ratio; the fibril length, *l*_1_; the total pixel number; and the orientation angle of the fibril relative to the direction of strain application, θ_1_, were recorded. The macroscopic strain applied to the PDMS membrane, ɛ, was calculated by measuring the length between PDMS clamp points in the strain device before and after application of strain or relaxation. The transverse compression due to anisotropy of the strain field, ɛ_T_, was measured macroscopically in each experiment by measuring the width of the stretchable membrane beneath the region of cell culture. Hence, the starting length of each individual fibril, *l*_0_, was calculated by

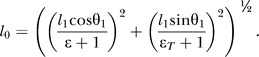
Finally, the estimated strain for each fibril, ɛ_fibril_, was calculated by

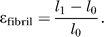



## Supporting Information

Figure S1 The Impact of Photobleaching and Intermolecular FRET on Measurements of Fn-DA in Fibrils (A) Fn-u and Fn-DA were run on SDS-PAGE gels and stained with Coomassie blue. Lane contents from left to right are as follows: Fn-u, amine/cys Fn-DA, empty, molecular weight ladder, Fn-u reduced in 50 mM DTT, and amine/cys Fn-DA reduced in 50 mM DTT.
(B) *I*_A_/*I*_D_ means ± standard deviations were measured for dimeric amine/cys Fn-DA in 0 (white), 1 (light gray), or 4 M GdnHCl (dark gray), where acceptor intensity varied according to Fn-DA concentration, laser transmissivity, and pixel dwell time. All images were acquired at 256 × 256 resolution for matched numbers of data points. Photobleaching was quantified by acquiring two images each of both acceptor (566–578 nm on PMT1) and donor (514–526 nm on PMT2) channels within a random field of view and plotting histograms of the differences in acceptor and donor intensities on a pixel by pixel basis between consecutive images.
(C–F) After averaging and background subtraction, the first-pass intensity was subtracted from the second-pass intensity in both acceptor (C and D) and donor (E and F) channels on a pixel by pixel basis. Intensity differences were plotted as histograms.
(G) Variable ratios of amine/cys Fn-DA (yellow circles) or cys/cys Fn-DA (blue diamonds) to Fn-u were added to culture medium of fibroblasts for 24 h. The total concentration of supplemented Fn remained constant at 50 μg/ml. Color-coded *I*_A_/*I*_D_ ratiometric images were acquired from three separate experiments at each ratio, and the mean and standard deviation of separate experiments at each ratio were plotted.
(3.9 MB TIF)

Figure S2 Impact of Noise Variance on Measurements of Fn-DA (A) To determine the impact of PMT background variability on *I*_A_/*I*_D_, all *I*_A_/*I*_D_ pixel measurements for a representative field of view within a denuded culture of cys/cys Fn-DA–containing fibrils were plotted versus donor intensity (filled circles). Standard deviations were calculated for all data points within bins with widths of 30 relative intensity units (yellow circles).
(B) The impact of the threshold on removal of highly variable *I*_A_/*I*_D_ measurements was tested by plotting the mean (yellow points), standard deviation (black bars), and 5% and 95% confidence intervals (black lines) for *I*_A_/*I*_D_ from a representative field of view within amine/cys Fn-DA–containing fibroblast matrix by excluding all data pixels with either acceptor or donor intensities below the threshold. The total number of data pixels remaining after application of the threshold mask is shown (blue circles).
(512 KB TIF)

Figure S3 Cys/cys Fn-DA in Matrix Fibrils (A–C) Cys/cys Fn-DA and excess Fn-u were added to culture medium of fibroblasts for 24 h. Color-coded *I*_A_/*I*_D_ ratiometric images are shown for control cells (A), extracted cell-free matrix (B), and fibroblast cells after 60 min of exposure to the ROCK inhibitor Y-27632 (C). Scale bars = 50 μm.
(D) Histograms were generated with 0.01-ratio-unit bin widths for all pixels of control (black), cell-free (purple), and ROCK-inhibited matrix (pink) derived from three random fields of view each from three separate experiments in each group. Orange, light blue, and dark blue lines are superimposed over the histograms and represent solution denaturation values for dimeric Fn-DA in 0, 1, and 2 M GdnHCl, respectively.
(1.5 MB TIF)

Figure S4 Schematic Diagram of the Strain Device The fully assembled strain device, shown upside down as a three-dimensional schematic for clarity (A), is composed of the main body (B) and end clamp pieces (C). All measurements are in millimeters. PDMS strips, 1.7 by 6 cm, were cut into rectangular or dog-bone shapes from 0.25-mm-thick sheets for surface modification and assembly into the device.
(860 KB TIF)

Figure S5 Strain Device Calibration To calibrate the degree of transverse compression relative to the magnitude of stretch applied to the PDMS membrane in the strain device, donor-labeled Fn was covalently linked to the PDMS substrate and photobleached into rectangular shapes. A 46 × 400 μm photobleached rectangle is shown before (A) and after (B) application of 38% strain. Height and width of rectangles were measured before and after stretch or relaxation of prestretched membranes, and linear regression of transverse compression versus elongation was used to verify that the strain field was uniform (C). Region of interest analysis was used to measure mean values for individual fibrils as described in Materials and Methods. A fluorescent donor image of a representative de-cellularized field of view of amine/cys Fn-DA–containing matrix is shown (D). Color-coded *I*_A_/*I*_D_ pixels within six analyzed fibrils are overlaid on the fluorescent donor image (E). Color-coded *I*_A_/*I*_D_ ratiometric images are shown for cell-free matrix assembled from 10% cys/cys Fn-DA without application of stretch (F) and after application of 65% elongation strain with 12% transverse compression (G). Individual fibrils were measured to determine the impact of elongation on *I*_A_/*I*_D_ on a per fibril basis (circles, mean ± standard deviation), and averages were calculated for fibrils without strain and fibrils that were strained from 26% to 72% (red squares, mean ± standard deviations) (H). Abscissa is also plotted as relative length change. Solution values for dimeric Fn-DA in 1 and 2 M GdnHCl are shown as horizontal light and dark blue lines, respectively. Scale bars = 50 μm.
(3.9 MB TIF)

Figure S6 Freely Detached Fibrils Derived from Substrate Stretch Cys/cys Fn-DA (A and B) or amine/cys Fn-DA (C and D) was incorporated into fibroblast matrix on plasma cleaned PDMS, and after cell extraction the substrate was stretched up to a 20% strain. *I*_A_/*I*_D_ ratiometric images of fully relaxed cys/cys Fn-DA–containing (A) or amine/cys Fn-DA containing (C) matrix fibrils are shown. Freely detached fibrils are shown within the white ovals, while still-attached fibrils are denoted with white arrows. Histograms for all pixels within the detached portion of the fibrils (white oval) are shown for cys/cys Fn-DA (B, mean ≈ 0.52) and amine-cys Fn-DA (D, mean ≈ 0.72). Scale bars = 50 μm.
(1.4 MB TIF)

Text S1 Fibronectin Isolation, Measurement Noise, and Strain Device Calibration and Usage (72 KB DOC)

Video S1 *Z*-Stack Overlay of the *I*_A_ */I*_D_ and DIC Images of Fibronectin Matrix in Fibroblast Culture Amine/cys Fn-DA and excess Fn-u were added to culture medium of fibroblasts for 24 h. Color-coded *I*_A_/*I*_D_ ratiometric images (A), transmitted DIC images (B), *I*_A_/*I*_D_ images overlaid on DIC images (C), and the histogram for all pixels within each *I*_A_/*I*_D_ image (D) are shown for a *z*-stack movie through typical field of view acquired using confocal microscopy with 1-μm increment steps in *z* from the top of the matrix towards the glass–cell interface. Solution denaturation values for dimeric Fn-DA in 0 M GdnHCl and monomeric Fn-DA in 1 and 4 M GdnHCl are shown as vertical red, blue, and green lines, respectively, over the histogram. Field of view dimensions are 106 × 106 μm.
(7.3 MB AVI)

## Abbreviations

amine/cys Fn-DA: amine/cysteine FRET-labeled fibronectin
CD: circular dichroism
cys/cys Fn-DA: cysteine/cysteine FRET-labeled Fn
DIC: differential interference contrast
DMEM: Dulbecco's modified Eagle's medium
DTT: DL-dithiothreitol
ECM: extracellular matrix
EDTA: ethylenediaminetetraacetic acid
Fn: fibronectin
Fn-DA: Fn labeled with Alexa 488 and Alexa 546
Fn-u: unlabeled Fn
FRET: fluorescence resonance energy transfer
FnIII: fibronectin type III
GdnHCl: guanidine hydrochloride
GFP: green fluorescent protein
*I*_A_*I*_D_: ratio of acceptor to donor intensities
NCS: newborn calf serum
PBS: phosphate-buffered saline
PDMS: poly(dimethylsiloxane)
PMT: photomultiplier tube
ROCK: Rho kinase
