# Supplementary material for: Force-Induced Unfolding of Fibronectin in the Extracellular Matrix of Living Cells
Source: PLoS Biol. 2007 Oct 2;5(10):e268. doi: 10.1371/journal.pbio.0050268 (PMC1994993; doi:10.1371/journal.pbio.0050268)
Supplement: Supplementary file 1 [file pbio.0050268.sd001.doc]

**TEXT S1**

***Fibronectin isolation from human plasma***

Human plasma Fn was isolated from fresh human plasma (Swiss Red Cross) using gelatin-sepharose chromatography based on established methods [1]. Briefly, 2 mM phenylmethylsulphonyl fluoride (PMSF) and 10 mM ethylenediaminetetraacetic acid (EDTA) were added to human plasma and spun at 15*10^3^ *g* for 40 min. Plasma was first passed over the gelatin Sepharose 4B column (Pharmacia) and subsequently the flow-through passed over the Sepharose 4B column (Sigma-Aldrich). The gelatin column was washed with 2 mM PMSF and 10 mM EDTA in PBS. Wash completion was verified when the 280 nm absorbance of the flow-through was <0.05. The gelatin column was washed with 1 M NaCl, 1 M urea, and finally Fn was eluted from the column with 6 M urea. Purity was approximated by silver stain and western blot. Isolated Fn was stored at -80°C in 6 M urea until usage.

***Fn-DA considerations***

This study employed a previously published FRET-based assay to infer the conformational states of both soluble and fibrillar Fn [2,3], however this technique was modified for use with a confocal microscope. Briefly, Fn was labeled for FRET using one of two labeling schemes and a reduction in energy transfer between donor (Alexa 488) and acceptor fluorophores (Alexa 546) indicates fluorophore separation and was used as an indirect indication of Fn extension [2,4]. Samples were excited using a 488 nm laser, and emitted donor and acceptor light were measured pixel by pixel in a left to right, top to bottom reading frame, and each pixel was detected simultaneously on two PMTs each equipped with a diffraction grating and slit. Using this approach each PMT was tuned to sample the donor and acceptor peak wavelength positions, and spatially resolved I_A_/I_D_ maps were constructed.

First, Fn-DA was investigated by Coomassie blue staining of samples run with a NuPAGE Large Protein Analysis System (Invitrogen) to determine whether the protein was completely reduced and if the isolated protein was contaminated by low molecular weight Fn fragments. Briefly, 0.5 μg of protein was loaded into each lane, and Fn-u and amine/cys Fn-DA were each analyzed with or without 60 minute incubation with 50 mM DTT (Fig. S1A). These results confirmed that Fn was fully reduced by 50 mM DTT and that significant quantities of Fn fragments were not present in isolated plasma Fn.

The specificity of labeling with maleimide-conjugated fluorophores was investigated by attempting to label only the amino-terminal 70 kDa fragment of Fn (obtained from Sigma), which does not contain the free cysteines located within modules III_7_ and III_15_, with Alexa 488 maleimide. The 70 kDa fragment was incubated with a 30-fold molar excess of Alexa 488-maleimide in 4 M GdnHCl in PBS and incubated for 1 hour. After overnight dialysis into PBS (Slide-a-lyzer dialysis cassette, 3500 MW cutoff; Pierce Biotechnology), the remaining Alexa 488 in the cassette was not above background. This indicates that maleimide conjugation of Fn is limited to within modules III_1-15_ and I_10-12_, suggesting that maleimide-conjugated fluorophores bind to III_7_ and III_15_.

Next, the confocal system was characterized to determine the range of confocal settings suitable for I_A_/I_D_ measurements. A 50/50 beamsplitter was used to divide emitted sample light onto each of two PMTs for simultaneous detection of both acceptor and donor intensities. Attenuation of light detected by PMT2 relative to PMT1 was quantified by measuring the donor peak intensity between 514 and 526 nm on both PMTs simultaneously for amine/cys Fn-DA in solution. Light detected on PMT2 was consistently ~92% of the intensity of light detected by PMT1 due to the presence of both the beamsplitter and a mirror in the light path of PMT2 as opposed to only the beamsplitter for light directed to PMT1, so for all subsequent measurements the donor intensity, measured on PMT2, was multiplied by a factor of 1.09 to compensate for this attenuation. Next the stability of I_A_/I_D_ values was measured over a wide range of relative intensity units by varying the solution concentration of amine/cys Fn-DA, the laser transmissivity, and the pixel dwell time. Figure S1B shows that dimeric amine/cys Fn-DA in 0, 1, and 4 M GdnHCl showed different I_A_/I_D_ values. In addition, within each group the I_A_/I_D_ values were very stable over a wide range of intensities above ~100 units, and therefore the wide ranging intensities between pixels within a fibroblast matrix field of view do not adversely affect I_A_/I_D_.

Confocal microscopy requires intense excitation power, and I_A_/I_D_ measurements are dramatically impacted if one fluorophore is more or less susceptible to photobleaching. A routine photobleaching test was developed for measurements of Fn matrix. Consecutive I_A_/I_D_ measurements were acquired for random fields of view and tested for a loss in intensity from the first scan to the second on a pixel by pixel basis. A histogram of percent intensity change from the first to second scan demonstrated significant damage to both acceptor (Fig. S1C) and donor fluorophores (Fig. S1D) within a representative field of view imaged at 45% excitation power and 20 μs dwell time. Although donors which form FRET pairs should be less susceptible to photodamage, the significant drop in donor intensity could also be due to photobleached donors which did not form FRET pairs, however this still impacts I_A_/I_D_ measurements since the donor background level is altered. Histograms from a different field of view in the same sample acquired with 5% excitation power and 10 μs dwell time showed no consecutive loss of fluorescent intensity of acceptors (Fig. S1E) and donors (Fig. S1F). For all I_A_/I_D_ measurements a mean loss of greater than 5% intensity for either the donor or acceptor was used to exclude fields of view which were compromised by photobleaching. When photobleaching did occur, color-coded spatial maps of acceptor or donor intensity differences in consecutively acquired images revealed that photobleaching was randomly distributed throughout the field of view and not localized to specific regions (data not shown), thus indicating that heterogeneous patterns of I_A_/I_D_ were not due to heterogeneous photobleaching.

Finally, FRET measurements would also be impacted if acceptors and donors on adjacent molecules of Fn-DA formed FRET pairs since the conclusions drawn in this study are based on the assumption that only intramolecular FRET occurs. Therefore, I_A_/I_D_ measurements of Fn-DA in fibroblast matrices were performed where the ratio of amine/cys or cys/cys Fn-DA to Fn-u was titrated over a wide range. An increase in measured I_A_/I_D_ with an increasing percentage of Fn-DA relative to unlabeled Fn would indicate that higher ratios of Fn-DA increases the likelihood of FRET pair formation between adjacent molecules of Fn-DA. In contrast, measurements of I_A_/I_D_ which were independent of the ratio of Fn-DA to Fn-u would indicate that only intramolecular FRET occurred. This analysis demonstrated that 10% amine/cys Fn-DA is not contaminated by intermolecular FRET (Fig. S1G). However, >10% amine/cys Fn-DA led to progressively increased mean I_A_/I_D_ values in fibrils. In contrast, mean I_A_/I_D_ measurements in ECM fibers using cys/cys Fn-DA were not dependent upon the ratio of cys/cys Fn-DA to Fn-u at ratios below 50% Fn-DA. This is consistent with the reduced number of fluorophores with this approach (~4) relative to amine/cys Fn-DA (~11).

***Impact of system noise in FRET measurements***

Acceptor and donor signals are acquired by separate PMTs, but only the mean PMT dark current value was used for background subtraction. Measurements of fibroblast ECM assembled only with Fn-u were similar to the mean PMT dark current, thus indicating that PMT noise was the predominant component of the background. The PMT dark current was on average 155 out of 4096 but ranged from 120 to 320 in a 512 by 512 pixel image. Therefore, the variability of measured I_A_/I_D_ values is predicted to become dramatically increased at low intensities as the ratio of noise variance to signal increases. Figure S2A shows a scatter plot for all I_A_/I_D_ pixels within a representative field of view as a function of donor intensity. In addition, the standard deviation of I_A_/I_D_ measurements within bins of width 30 relative intensity units was calculated. Figure S2A shows that I_A_/I_D_ deviation initially drops precipitously up to ~100 relative intensity units, a finding which was representative for all measured fields of view. To further characterize the impact of dim pixels on I_A_/I_D_ measurements, a representative field of view containing amine/cys Fn-DA was analyzed using different threshold levels where pixels with either acceptor or donor intensities below the plotted threshold value were excluded from analysis. A plot of mean, standard deviation, and 5 and 95% confidence intervals for all data within the field of view above the intensity threshold shows that the I_A_/I_D_ measurements stabilize above ~100 relative intensity units above background, however the total number of data pixels is significantly reduced as the threshold value increases further (Fig. S2B). For these reasons a threshold value of 100 relative intensity units was used in both donor and acceptor channels for all measurements in fibrils. Similar results were found using cys/cys Fn-DA in culture (data not shown).

***Cys/cys Fn-DA in culture***

To test for the presence of the fully compact solution structure of Fn, cys/cys Fn-DA was incorporated into a 24 hour fibroblast ECM to test for the presence of the fully compact solution structure of Fn. Figure S3A shows a ratiometric confocal slice 4 μm above the glass/ECM interface. Cell extraction with Triton X-100 (Fig. S3B), which led to relaxation of the ECM into a more 2D structure that was generally less than 4 μm in thickness, and Rho kinase (ROCK) inhibition with Y-27632 (Fig. S3C), which led to cell rounding and fewer cell extensions, were used to inhibit cellular contractile forces and therefore cause matrix relaxation [3,5]. Histograms for all pixels in three random fields of view each from three separate experiments are plotted in Fig. S3D. Some regions of cyc/cys Fn-DA matrix were composed of Fn with partial backfolding since I_A_/I_D_ values were higher than that measured for the extended structure but lower than that measured for the fully compact solution structure. These data confirm that the compact solution structure of Fn was never achieved in fibrillar Fn even after release of cell traction forces.

***Strain device assembly and calibration***

A strain device was designed for application of known quantities of strain to a PDMS substrate according to dimensions in Fig. S4. The device was manufactured from stainless steel. PDMS membranes could be surface functionalized with either adsorbed or covalently attached Fn and subsequently clamped into the strain device. In order to quantify uniformity of the strain field and determine the quantity of transverse compression relative to the direction of applied strain, Fn labeled only with donor fluorophores was covalently attached to the membranes. Various shapes such as rectangles were photobleached into the surface bound PDMS, the strain device was used to stretch the PDMS membrane by known quantities, and the photobleached shapes were re-imaged. Figures S5A and B show a photobleached rectangle imaged prior to strain (Fig. S5A) and after 38% elongation strain of the PDMS membrane (Fig. S5B). Figure S5C shows the relationship between elongation and transverse compression for one such rectangular shaped specimen demonstrating that the PDMS substrate had a linear relationship between elongation and transverse compression. For this PDMS specimen, transverse shortening of ~40% of the magnitude of elongation occurred. The degree of transverse shortening could be reduced by cutting dog bone shaped specimens. For all strain device experiments, the extent of transverse shortening relative to the extent of elongation was measured macroscopically under the area of cell culture on the PDMS membrane.

After surface functionalization of Fn-u to the PDMS membrane, fibroblast cells assembled an Fn matrix from Fn-u and Fn-DA in solution. Cells were subsequently removed with mild detergent leaving behind only the Fn-DA-containing matrix attached to the stretchable PDMS membrane in order to eliminate a cellular response to strain. Although the PDMS sheet is elongated by a known quantity, individual fibrils are stretched or relaxed by different amounts depending on their orientation relative to the direction of stretch (see Methods). Therefore mean I_A_/I_D_ was measured for individual fibrils rather than the entire field of view (Figs. S5D, E). Elongation of the PDMS sheet was limited to less than 1.75-fold increase in length (75% strain) since the cell-free matrix generally detached from the surface under greater strain (data not shown). Individual fibril mean I_A_/I_D_ values were next determined for matrix attached to the PDMS without membrane stretch or after elongation of PDMS membranes or relaxation of membranes which were stretched prior to cell seeding. Fibrils were chosen which met criteria outlined in Methods. Figure S5D shows the donor channel image of a denuded amine/cys Fn-DA-containing matrix on a PDMS membrane prior to stretch, and Fig. S5E shows I_A_/I_D_ color-coded for 6 fibrils which were analyzed using region of interest analysis.

Next, cell-free matrix containing 10% cys/cys Fn-DA was stretched to determine whether cys/cys Fn-DA was strain responsive. Relative strain of individual fibrils ranged from 1.26- (26% strain) to 1.72-fold stretch (72% strain). Cell-free control fibrils measured without strain had mean I_A_/I_D_ of 0.46±0.04 (n=29 fibrils), and I_A_/I_D_ mean values for individual fibrils ranged from 0.39 to 0.54. Cell-free fibrils which were stretched by 53±14% strain had mean I_A_/I_D_ of 0.45±0.03 (n=33 fibrils) which was not statistically significant from control fibrils. These data indicate that cys/cys Fn-DA is incapable of differentiating extended from unfolded Fn and also confirms that intermolecular FRET does not impact measurements with cys/cys Fn-DA. However, it should be noted that the range of I_A_/I_D_ mean values for individual stretched fibers was narrower, from 0.39 to 0.50, than the total range of measured values which were not subjected to strain. This likely reflects that a few cys/cys Fn-DA cell-free fibers were in partially backfolded conformations which permitted minor energy transfer, and this contribution to energy transfer was lost after mild fiber stretching.

***Fully relaxed matrices***

To test whether Fn assumes a compact conformation in the resting state of fibrils [6], fibroblast matrix composed of 10% cys/cys Fn-DA or amine/cys Fn-DA was grown on Fn-u adsorbed onto PDMS sheets. Without covalent attachment of Fn-u to PDMS, 10-20% strain resulted in detachment of large regions of now fully relaxed de-cellularized ECM with relatively homogeneous conformation (Fig. S6). These aggregates of Fn fibrils were loosely attached to the membrane, randomly diffused around their remaining points of attachment to the substrate, and often extended vertically for up to 10 μm away from the membrane. Although strain application was required for detachment from the substrate, subsequent relaxation did not result in an increase in I_A_/I_D_ of these structures, again indicating that detached fibrils were fully relaxed. I_A_/I_D_ mean values for fully relaxed fibrils containing cys/cys Fn-DA (Fig. S6B) or amine/cys Fn-DA (Fig. S6D) varied from 0.48 to 0.58 and 0.68 to 0.75, respectively. The percentage of fully compact pixels never exceeded 1% using cys/cys Fn-DA, while at most 6% of pixels were fully compact using amine/cys Fn-DA. Considering the boundary condition that a 3.5-fold length change can occur from transition between fully compact and extended conformations [6], Fn molecules in fully relaxed fibrils which are already elongated relative to the compact conformation must unfold in order to accommodate 4-fold length changes estimated using Fn-GFP proteins [5,7]. This provides additional evidence that Fn must be unfolded in matrix fibrils regardless of the solution I_A_/I_D_ considered to be the unfolding point.

**References**

1. Engvall E, Ruoslahti E (1977) Binding of soluble form of fibroblast surface protein, fibronectin, to collagen. Int J Cancer 20: 1-5.

2. Baneyx G, Baugh L, Vogel V (2001) Coexisting conformations of fibronectin in cell culture imaged using fluorescence resonance energy transfer. Proc Natl Acad Sci U S A 98: 14464-14468.

3. Baneyx G, Baugh L, Vogel V (2002) Fibronectin extension and unfolding within cell matrix fibrils controlled by cytoskeletal tension. Proc Natl Acad Sci U S A 99: 5139-5143.

4. Baugh L, Vogel V (2004) Structural changes of fibronectin adsorbed to model surfaces probed by fluorescence resonance energy transfer. J Biomed Mater Res 69A: 525-534.

5. Ohashi T, Kiehart DP, Erickson HP (2002) Dual labeling of the fibronectin matrix and actin cytoskeleton with green fluorescent protein variants. J Cell Sci 115: 1221-1229.

6. Abu-Lail NI, Ohashi T, Clark RL, Erickson HP, Zauscher S (2006) Understanding the elasticity of fibronectin fibrils: unfolding strengths of FN-III and GFP domains measured by single molecule force spectroscopy. Matrix Biol 25: 175-184.

7. Ohashi T, Kiehart DP, Erickson HP (1999) Dynamics and elasticity of the fibronectin matrix in living cell culture visualized by fibronectin-green fluorescent protein. Proc Natl Acad Sci U S A 96: 2153-2158.
